# Online Patient Reviews for Continuous Quality Improvement: Topic Modeling of Hospital Service Quality in Taiwan and the United States

**DOI:** 10.3390/healthcare14111580

**Published:** 2026-06-04

**Authors:** Sheng-Hsun Hsu, Shwu-Fen Chiu

**Affiliations:** 1 NTHU Center for Continuing Education, National Tsing Hua University, Hsinchu 300044, Taiwan; shenghsun@mx.nthu.edu.tw; 2 Department of Business Administration, Chung Hua University, Hsinchu 30012, Taiwan; 3 President’s Office, Dachien General Hospital, Miaoli 36052, Taiwan

**Keywords:** continuous quality improvement, patient experience, healthcare service quality, topic modeling, Latent Dirichlet Allocation, online reviews, HCAHPS, cross-national comparison

## Abstract

**Background/Objectives**: Continuous quality improvement (CQI) requires timely, patient-centered evidence on how people experience healthcare delivery. Structured surveys provide important benchmarks, but their predetermined items may miss emerging or system-specific concerns. This study assesses whether unsolicited online patient reviews can serve as a scalable patient-experience data source for identifying hospital service quality priorities across contrasting healthcare systems. **Methods**: We analyzed 8247 Google Maps hospital reviews posted in 2024, including 5007 Chinese-language reviews from 24 Taiwanese medical centers and 3240 English-language reviews from 21 large U.S. referral hospitals. Separate language-specific preprocessing pipelines and Latent Dirichlet Allocation (LDA) topic models identified patient-salient service quality dimensions in each country. Cross-lingual semantic mapping then distinguished universal dimensions from system-specific concerns, and star-rating differences across semantically equivalent dimensions were compared. **Results**: Seven service quality dimensions emerged in each country: five were cross-nationally shared (emergency care, positive care experience, professional medical team, administrative process, and inpatient/treatment care), and each system had two system-specific dimensions. Taiwanese reviews foregrounded service attitude and facility/environment quality, while U.S. reviews foregrounded billing/insurance and clinic systems/access. Ratings for emergency care and administrative process were consistently low across both systems, whereas ratings for the professional medical team were substantially higher in U.S. reviews. **Conclusions**: Online patient reviews can complement formal patient-experience instruments by revealing actionable CQI priorities that are both universal and context dependent. Emergency care and administrative efficiency represent shared improvement needs across both systems. System-specific interventions include interpersonal training and infrastructure investment in high-utilization single-payer settings, and billing transparency and care coordination in fragmented multi-payer systems. Institutional structures appear to play a more prominent role than cultural factors in shaping which service quality dimensions emerge, though both forces contribute. Established frameworks may inadequately capture system-specific patient concerns.

## 1. Introduction

Continuous quality improvement (CQI) in hospitals depends on timely evidence about how patients experience care delivery. Service quality research provides a foundation for this patient-centered view: SERVQUAL conceptualizes perceived service quality as the gap between expectations and performance and identifies reliability, responsiveness, assurance, empathy, and tangibles as core dimensions [1,2]. Healthcare differs from most consumer services, however, in that patients typically cannot directly assess technical quality, clinical appropriateness, or long-term outcomes [3,4]. Patients instead rely on observable cues—interpersonal communication, environmental conditions, administrative efficiency, and perceived professional competence—when forming quality judgments [5,6]. Because these cues correspond to modifiable operational processes, they are directly relevant to CQI.

Formal patient-experience instruments, including the Hospital Consumer Assessment of Healthcare Providers and Systems (HCAHPS), Patient-Reported Experience Measures (PREMs), SERVQUAL-derived scales, and patient satisfaction surveys, operationalize these ideas for hospital benchmarking and accountability. Yet structured instruments impose predefined categories, are susceptible to cross-cultural response-style differences, and may miss concerns that survey designers did not anticipate [7,8,9]. These limitations are particularly consequential for cross-national CQI, where identical items may not carry equivalent meaning across institutional and cultural contexts.

Online patient reviews offer a complementary data source that captures patient experience in patients’ own terms. Reviews are unsolicited, narrative, and often emotionally salient, and prior evidence shows that hospital ratings and review narratives on major platforms contain patient-experience signals that align meaningfully with established quality measures [10,11]. The analytical challenge lies in converting these unstructured narratives into interpretable quality domains. Topic modeling, particularly Latent Dirichlet Allocation (LDA), addresses this challenge by inductively recovering latent themes from large text corpora without imposing predefined categories [12,13]. For multilingual comparisons, running language-specific LDA models and mapping topics across languages preserves language-specific meaning while enabling cross-national interpretation [14,15].

Recent advances in natural language processing (NLP) offer alternative approaches to patient-experience analysis. Transformer-based models such as BERT provide contextual word embeddings [16], and BERTopic uses such embeddings to support topic modeling with semantically informed document representations [17]. Aspect-based sentiment analysis (ABSA) can further extract sentiment toward specific service attributes [18]. A growing body of recent work applies these methods to healthcare contexts: Dai et al. [19] combined LDA with sentiment analysis to evaluate medical service quality across chronic disease categories; Placona and Rathert [20] found that online patient reviews are associated with patient experience and, more variably, with healthcare quality outcomes; and Khanbhai et al. [21] systematically reviewed NLP and machine learning applications to patient experience feedback. However, supervised and semi-supervised approaches require labeled training data that may not be available across languages and healthcare contexts. For inductive, cross-national comparison—where the research objective is to discover which service quality dimensions emerge without imposing predetermined categories—unsupervised LDA remains well suited because it requires no labeled data, produces interpretable topic structures for CQI practitioners, and allows independent modeling of each language corpus before cross-lingual mapping.

Despite this growing methodological toolkit, most existing studies apply topic modeling within a single country or language [14,19,21], and few have attempted systematic cross-lingual semantic mapping to distinguish universal from system-specific patient concerns. This study addresses that gap by combining independent language-specific LDA with a structured cross-lingual alignment protocol.

Taiwan and the United States represent a theoretically informative paired comparison because they share high levels of healthcare capacity and digital connectivity but differ fundamentally in financing structure and cultural orientation. Taiwan’s single-payer National Health Insurance provides universal coverage with minimal financial barriers, whereas the U.S. system features fragmented insurance arrangements, substantial cost sharing, and administrative complexity [22,23]. Both countries have substantial Google Maps hospital review volumes in their respective primary languages, enabling independent language-specific analysis. These institutional differences may shape which service dimensions patients find most salient: high utilization and service-flow pressures in Taiwan may heighten attention to interpersonal treatment, patient flow, and facility strain [24,25], whereas insurance complexity, billing uncertainty, and care-coordination burdens may make financial and administrative concerns more salient in U.S. patient evaluations [26,27].

The two countries also differ culturally: Taiwan lies toward the collectivist end of Hofstede’s cross-cultural dimensions, whereas the United States lies toward the individualist end [28]. In collectivist settings, the interdependent self emphasizes relational harmony and attentiveness to others [29], which may help explain why interpersonal demeanor functions as a central quality criterion. Taiwan also scores higher on power distance [28], suggesting that patient deference to physician authority may coexist with expectations for respectful treatment in service encounters. Individualist cultures, by contrast, tend to place greater emphasis on autonomy and individually differentiated expectations, and prior service-quality research indicates that cultural orientation shapes how consumers weight service-quality dimensions [30]. These cultural orientations may shape not only which service attributes patients notice but also whether specific concerns coalesce into standalone evaluative dimensions or remain embedded within broader experience categories. Institutional and cultural factors therefore jointly condition which evaluative domains surface in patient narratives.

Prior cross-national studies comparing patient-experienced service quality dimensions in these two healthcare systems remain scarce. Using structured patient-experience surveys, Yoon and Cheng [31] documented higher perceived inpatient care quality in the United States and opposite socioeconomic gradients in Taiwan and the United States. Because that comparison relied on predefined survey dimensions, however, it could not determine whether each healthcare system generates its own emergent quality concerns. More broadly, existing studies have not established whether patients in fundamentally different systems emphasize the same service-quality domains or whether each system produces distinct improvement priorities. This gap is well suited to inductive analysis of unsolicited patient narratives.

This study analyzes 5007 Chinese-language reviews from 24 Taiwanese medical centers and 3240 English-language reviews from 21 large U.S. referral hospitals using parallel LDA pipelines, then maps the derived dimensions cross-lingually to separate universal CQI priorities from system-specific concerns. Three research questions guide the analysis: (1) What patient-experienced service quality dimensions emerge in each healthcare system? (2) Which dimensions are shared across systems, and which are system-specific? (3) How do institutional and cultural contexts shape these cross-national patterns? By using unsolicited patient narratives as a supplementary patient-experience signal, the study contributes to healthcare CQI research in three ways: it identifies universal quality concerns that support cross-national learning; it surfaces system-specific concerns that require locally tailored interventions; and it demonstrates how topic modeling can translate large-scale patient narratives into actionable CQI priorities for hospital managers and policymakers.

## 2. Materials and Methods

This study implemented a multi-stage analytical framework to inductively extract and compare patient-salient service quality dimensions from Taiwan and U.S. hospital reviews. The framework consisted of four stages: (1) data collection from Google Maps (Google LLC, Mountain View, CA, USA; https://www.google.com/maps, accessed on 7 January 2026), (2) language-specific text preprocessing, (3) independent LDA topic modeling for each country, and (4) cross-lingual semantic mapping to identify universal and system-specific dimensions.

### 2.1. Data Collection

Patient reviews were retrieved from Google Maps because the platform provides (1) consistent global coverage, (2) verified linkage between reviews and specific hospital locations, and (3) a standardized 1–5 star rating structure. Reviews were collected using automated scripts that retrieved publicly displayed review content via the Google Maps platform. The study analyzed only publicly available, anonymized user-generated content; no private, restricted, or personally identifiable information was accessed, and all data were used solely for non-commercial academic research purposes. Reviews were collected for the 2024 calendar year and retained only if they contained non-empty textual content.

#### 2.1.1. Taiwan Hospital Sampling and Review Retrieval

We focused on medical centers, the highest tier in Taiwan’s accreditation system, to capture evaluations of tertiary-level care. All 24 medical centers recognized by the Ministry of Health and Welfare were included. These institutions deliver complex care and account for a large share of nationwide tertiary service volume.

Using automated scripts, we retrieved Google Maps reviews during the data collection period and retained those whose derived posting dates fell between 1 January and 31 December 2024. Across the sampled hospital listings, the scraper returned 8547 review records. We excluded reviews with missing or empty text (including star-only ratings; n = 2833), yielding 5714 reviews with text. We then excluded reviews with no meaningful content after cleaning (e.g., emoji-only or symbol-only entries, or fewer than 5 characters; n = 431), yielding 5283 reviews. After word segmentation and stopword removal, we excluded reviews with fewer than 3 remaining tokens (n = 276). The final Taiwan corpus consisted of 5007 Chinese-language reviews.

#### 2.1.2. U.S. Hospital Sampling and Review Retrieval

For the U.S. sample, we targeted large referral hospitals as functional counterparts to Taiwan’s medical centers. We began with the U.S. News & World Report Best Hospitals Honor Roll (2024–2025 edition). Because several Honor Roll listings had limited Google Maps review volumes, we supplemented the sample with three additional high-volume referral hospitals (AdventHealth Orlando, Hackensack University Medical Center, University of Michigan Health) to ensure sufficient review volume for cross-national comparison. After consolidating multiple Google Maps listings, the final U.S. sample comprised 21 hospitals (18 Honor Roll + 3 additional).

All reviews were retrieved using the same automated procedure, and we retained those whose derived posting dates fell between 1 January and 31 December 2024. Across the included hospital listings, the scraper returned 5258 review records. We excluded reviews with missing or empty text (n = 1895), leaving 3363 reviews. We then restricted the analysis to English-language reviews (n = 123 excluded), yielding 3240 reviews. As with the Taiwan corpus, the U.S. corpus underwent the same subsequent text-cleaning and preprocessing checks to remove non-substantive reviews. No additional U.S. reviews were removed at this stage, so the final U.S. corpus consisted of 3240 English-language reviews.

### 2.2. Text Preprocessing

Separate preprocessing pipelines were applied to the Chinese and English corpora to remove noise. All preprocessing steps were executed prior to topic modeling.

#### 2.2.1. Chinese Preprocessing Procedures

Chinese reviews were first cleaned to remove HTML artifacts and URLs. Word segmentation was performed using the Jieba Python package (v0.42.1) with a custom domain dictionary to accurately tokenize multi-character medical terms. Stopwords were removed using a Chinese stopword list, supplemented with manually identified high-frequency non-informative terms. To ensure substantive preservation, two researchers manually inspected a random subsample to verify that key service-quality terms remained intact.

#### 2.2.2. English Preprocessing Procedures

English-language reviews were converted to lowercase, stripped of punctuation, numeric characters, and URLs, and then tokenized. Standard English stopwords and high-frequency non-discriminative terms were removed, while medically salient vocabulary (for example, emergency, pain, wait) was retained. A pair of researchers manually validated a subsample to verify that domain-relevant content was preserved.

### 2.3. LDA Modeling

**Figure 1:**
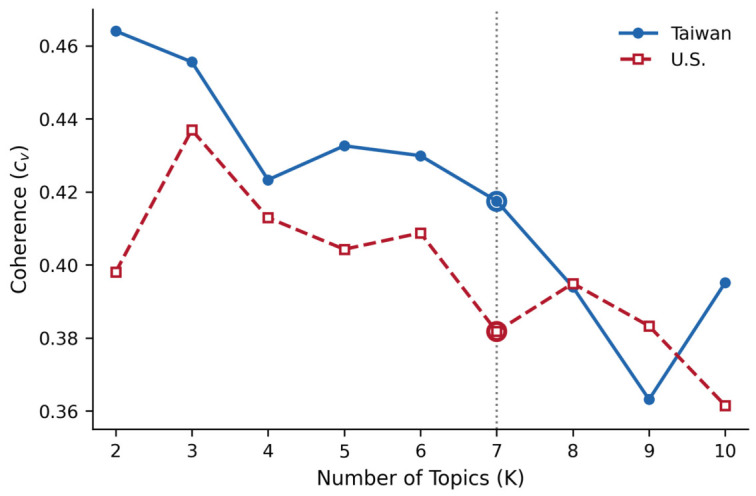
Topic coherence (c_v_) for candidate LDA models with K = 2–10 topics. The vertical dotted line marks the selected model (K = 7), and the large circles highlight the coherence values at the selected number of topics for each country.

LDA models were implemented in Python (v3.10.19) using Gensim (v4.4.0). For each corpus, tokenized reviews were converted into bag-of-words representations, and extreme terms were removed before model fitting. Candidate models with K = 2–10 topics were evaluated using the c_v_ coherence metric (Table 1, Figure 1). Coherence was used as a diagnostic indicator rather than as the sole selection rule, because lower-K models can achieve high coherence by merging conceptually distinct service-quality domains into broad topics.

Although the highest coherence values occurred at K = 2 or 3 in both corpora (Table 1), these solutions were too coarse to distinguish clinically meaningful service quality domains. At K = 6, billing and insurance concerns in U.S. reviews were absorbed into a broader administrative topic; at K = 8, the Taiwan solution began to fragment positive care experience into less clearly differentiated sub-topics. We therefore selected K = 7 to balance semantic coherence, topic interpretability, and cross-national comparability. Retaining K = 7 for both countries also allowed Taiwan and U.S. dimensions to be compared at the same topic granularity. Each review was assigned to its dominant topic based on the highest posterior topic probability, and topic-level proportions and mean star ratings were calculated for subsequent cross-national comparison.

### 2.4. Cross-Lingual Semantic Mapping

After independent LDA modeling, we performed cross-lingual semantic mapping to determine which dimensions were universal and which were system-specific. Two bilingual researchers independently evaluated each of the 49 possible Taiwan–U.S. topic pairs (7 × 7). Classification was based on overlap and conceptual correspondence among the top 20 keywords, with Taiwan keywords translated into English to facilitate cross-language comparison, and on thematic review of representative high-probability reviews from each topic. Topic pairs were classified as High similarity when keyword overlap and thematic content converged on the same service-quality domain, Medium when partial keyword overlap was present but thematic focus diverged, and Low when no meaningful correspondence was identified. Discrepant classifications were resolved through discussion until full consensus was reached. Only High-similarity dimensions were retained for quantitative rating comparisons.

### 2.5. Cross-National Statistical Comparisons

Cross-national comparisons proceeded in two steps. First, a presence and absence analysis identified dimensions that appeared in only one corpus, indicating system- or culture-specific concerns. Second, for topic pairs with high semantic similarity, independent-samples *t*-tests compared mean review ratings, with effect sizes reported using Cohen’s *d*. These tests were restricted to semantically equivalent dimensions to avoid invalid comparisons.

## 3. Results

This section reports the empirical findings from the cross-national analysis. Section 3.1 presents the seven LDA-derived service quality dimensions for each country. Section 3.2 provides cross-national comparisons based on semantic similarity classifications. Section 3.3 examines dimension-level presence–absence patterns and rating differences for High-similarity topics.

### 3.1. Service Quality Dimensions by Country

#### 3.1.1. Taiwan’s Seven-Dimensional Structure

LDA analysis with K = 7 revealed seven distinct service quality dimensions in Taiwan hospital reviews. Table 2 presents the complete dimensional structure with topic proportions, average ratings, and characteristic keywords.

Emergency care (T7: 30.9%, 1.79 stars) dominates Taiwanese patient discourse yet receives the lowest satisfaction rating. This suggests that Taiwan’s single-payer NHI system faces severe challenges in managing emergency department demand. In contrast, positive care experience (T1: 27.2%, 4.67 stars) represents the second-largest dimension and receives the highest rating. Examination of high-probability reviews reveals that patients describe physicians and nurses as technically competent, kind, and deeply committed to their well-being, frequently thanking specific doctors and nursing teams by name.

Service attitude problems (T3: 17.3%, 1.69 stars) emerge as a distinct dimension, indicating Taiwanese patients’ heightened sensitivity to perceived impoliteness or impatience from healthcare workers. High-probability reviews describe registration staff cutting patients off mid-sentence and nurses raising their voices to older adults.

Facility and environment quality (T4: 8.1%, 2.73 stars) addresses infrastructure strain under high utilization. Reviews describe crowded parking garages, long elevator waits, confusing internal routes, and restroom cleanliness problems.

Registration and billing process (T2: 6.9%, 1.83 stars) captures administrative inefficiencies including long queues at multiple service counters and fragmented flows between registration, billing, and pharmacy.

Surgical and specialty care (T5: 5.3%, 4.02 stars) receives positive ratings as patients express gratitude for successful surgeries and specialized medical procedures, highlighting the quality of surgical teams and specialty departments. Inpatient care experience (T6: 4.3%, 2.35 stars) addresses ward-level concerns including visitation policies, discharge planning, and day-to-day nursing care during hospitalization.

#### 3.1.2. U.S. Seven-Dimensional Structure

LDA analysis with K = 7 revealed seven distinct dimensions in U.S. hospital reviews. Table 3 presents the complete dimensional structure.

Emergency care and waiting-time complaints form a large, predominantly negative dimension (U1: 23.4%, 2.02 stars). Reviews describe prolonged emergency department waits, hallway boarding, and difficulty obtaining timely nursing attention. In contrast, overall positive care experiences (U2: 26.7%, 4.84 stars) capture an overwhelmingly positive halo around hospital care. Reviews show that patients praise nurses, staff, and physicians, emphasizing kindness, professionalism, and coordinated teamwork.

Clinic systems and access (U3: 7.8%, 2.60 stars) focuses on large integrated systems such as Cleveland Clinic and Mayo Clinic, highlighting mixed experiences with prestige, referral pathways, and fragmented coordination across sites. The professional medical team dimension (U4: 15.1%, 4.60 stars) concentrates praise on surgical and procedural teams, emphasizing technical competence, well-orchestrated procedures, and confidence in medical decision-making. Treatment and pain management (U5: 12.5%, 2.11 stars) aggregates negative stories about chronic illness mismanagement, diagnostic delays, and inadequate pain control.

Billing and insurance issues (U6: 4.1%, 2.40 stars) isolate complaints about surprise bills, insurance denials, prior authorization delays, and billing errors. Appointment and scheduling (U7: 10.4%, 2.00 stars) captures long waits for appointments, frequent rescheduling, and delayed test results, with patients describing months-long delays for specialist care.

### 3.2. Cross-National Comparison: Universal and System-Specific Dimensions

Systematic semantic mapping classified dimension pairs by similarity: High, Medium, or Low. This classification enables identification of universal concerns versus system-specific patterns.

**Figure 2:**
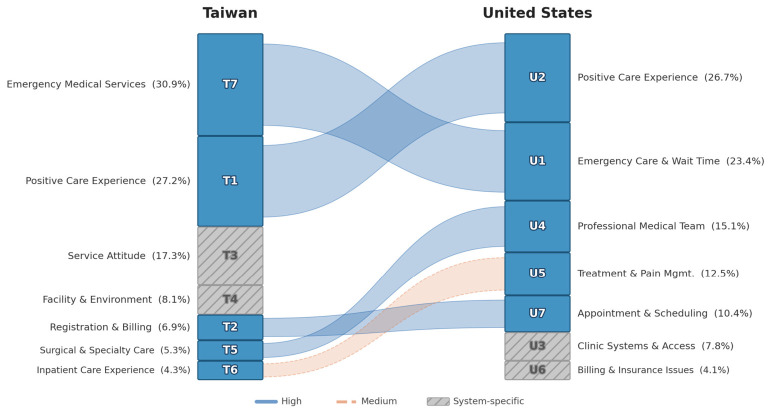
Cross-lingual semantic mapping of Taiwan and U.S. hospital service quality dimensions. Lines connect dimension pairs classified by similarity level (blue = High, orange = Medium). Hatched dimensions are system-specific (present in only one country).

Table 4 and Figure 2 present the semantic mapping results, revealing five universal dimensions common to both countries. Four dimension pairs demonstrate High similarity, while one pair (Inpatient/Treatment Care) shows Medium similarity.

**Figure 3:**
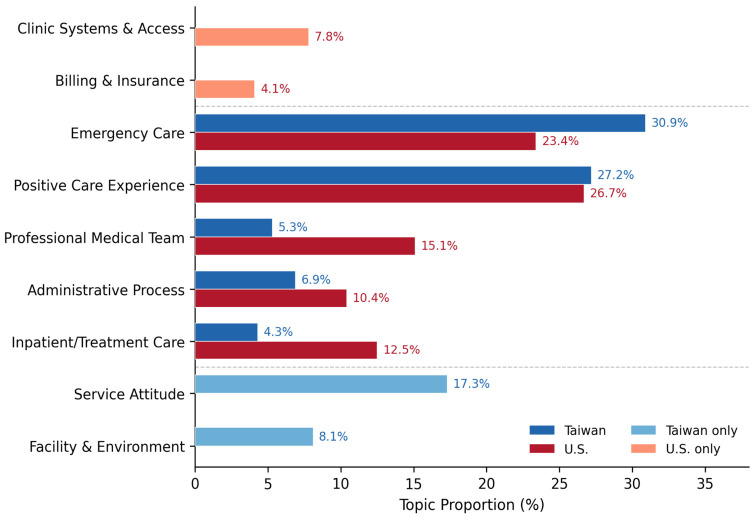
Cross-national comparison of topic proportions. Universal dimensions appear in both corpora; system-specific dimensions appear in only one country.

Figure 3 further provides a visual overview of topic proportions across both countries, distinguishing universal dimensions (present in both corpora) from system-specific dimensions (present in only one).

The five universal dimensions and four system-specific dimensions are discussed in detail below.

#### 3.2.1. Universal Dimensions

Emergency care represents the clearest universal concern, comprising approximately one-quarter to one-third of reviews in both countries with similarly low satisfaction. Patients across both systems describe overcrowded emergency departments, extended waiting times, and difficulties obtaining timely attention, suggesting that emergency services represent a shared structural vulnerability.

Positive care experiences constitute the largest or second-largest dimension in both systems, capturing broad gratitude toward healthcare professionals. Keywords in both topics emphasize care quality, professionalism, compassion, and appreciation.

The Professional Medical Team dimension receives high satisfaction in both contexts, though it is substantially more prominent in U.S. reviews. The Administrative Process dimension generates comparable dissatisfaction despite different manifestations. Taiwan patients complain about on-site queuing at registration counters and inefficient flows between registration, billing, and pharmacy services. U.S. patients describe months-long appointment delays and difficulties receiving test results. While the specific manifestations differ, the fundamental complaint remains consistent: the temporal burden of accessing and navigating the healthcare system.

The inpatient/treatment care pair achieves only Medium similarity because Taiwan reviews emphasize routine ward logistics, visitation policies, and discharge planning, while U.S. reviews focus on treatment plan failures, pain management issues, and chronic disease management, representing partially overlapping but distinct constructs.

#### 3.2.2. System-Specific Dimensions

Four dimensions emerge in only one country, providing evidence of cultural and institutional specificity. Table 5 summarizes these system-specific patterns.

These presence/absence patterns reveal how system design and cultural values might shape patient evaluations. Section 3.3.2 provides detailed institutional and cultural explanations for each dimension.

### 3.3. System and Cultural Effects

This section presents statistical evidence for system and cultural effects through two strategies: (1) rating comparisons via *t*-tests for High-similarity dimension pairs, and (2) institutional and cultural explanations for dimension presence/absence patterns.

#### 3.3.1. Rating Comparisons for High-Similarity Dimensions

For the four High-similarity dimension pairs identified in Table 4, we conducted independent samples *t*-tests comparing mean ratings between countries. The Inpatient/Treatment Care pair was excluded from statistical comparison because its Medium similarity indicated partial divergence in construct meaning, making direct rating comparisons invalid. Table 6 presents these comparisons.

Three comparisons reach statistical significance. Emergency Care and Positive Care Experience demonstrate small effects (*d* = −0.16 and −0.18), suggesting that despite statistically significant differences, both countries face similarly severe emergency care challenges and achieve comparable levels of satisfaction in positive care experiences. Professional Medical Team exhibits a medium effect (*d* = −0.52), suggesting that U.S. hospitals demonstrate a meaningful advantage in specialized procedural care. Administrative Process showed no significant difference (*p* = 0.117), suggesting comparable dissatisfaction across systems.

#### 3.3.2. Presence/Absence Patterns: Institutional and Cultural Effects

Four dimensions appear in only one country (Table 5), suggesting how institutional structures and cultural values may shape service quality evaluations. Three dimensions appear to reflect institutional differences in healthcare system design, while one appears more closely linked to cultural evaluative priorities.

(a) *Institutional Effects*

Billing & Insurance Issues (4.1%, 2.40 stars) appears as a distinct U.S. dimension with no Taiwanese counterpart. Taiwan’s Registration and Billing Process dimension captures administrative queues and service flow inefficiencies but contains no cost- or insurance-related keywords. This presence/absence pattern suggests that multi-payer financing may generate patient-facing financial complexity that single-payer coverage largely eliminates from quality evaluations.

Clinic Systems & Access (7.8%, 2.60 stars) emerges only in the United States, reflecting experiences with large integrated delivery systems such as Cleveland Clinic and Mayo Clinic. Reviews discuss system reputation, fragmented coordination across multiple sites, and organizational access barriers. Taiwan’s medical centers function as standalone institutions, so patients do not encounter comparable multi-site navigation challenges.

Facility & Environment Quality (8.1%, 2.73 stars) appears only in Taiwan, addressing parking shortages, crowded spaces, elevator waits, and cleanliness concerns. This dimension reflects infrastructure strain from high-utilization patterns in Taiwan’s unrestricted-access single-payer system.

(b) *Cultural Effects*

Service Attitude (17.3%, 1.69 stars) emerges as Taiwan’s third-largest and lowest-rated dimension with no standalone U.S. equivalent. Taiwanese patients devote an entire dimension to perceived rudeness, impatience, and disrespect from staff, indicating that interpersonal demeanor constitutes a core quality outcome rather than a secondary process detail. In U.S. reviews, staff attitude concerns appear embedded within broader emergency care, treatment quality, and overall experience dimensions. This asymmetry is consistent with Taiwan’s collectivist, relationship-oriented cultural orientation emphasizing interpersonal harmony and respect [28,29].

## 4. Discussion

This study compared how patients in Taiwan and the U.S. evaluate hospital service quality using LDA topic modeling and cross-lingual semantic alignment applied to online reviews. The following subsections summarize the key findings, articulate their theoretical and practical implications, and acknowledge limitations that frame directions for future research.

### 4.1. Principal Findings

The findings are organized around the three research questions that guided the analysis.

*RQ1: What patient-experienced service quality dimensions emerge in each healthcare system?* Applying LDA independently to 5007 Taiwanese and 3240 U.S. hospital reviews, we identified seven service quality dimensions in each country. In Taiwan, the seven dimensions were Emergency Medical Services (T7: 30.9%, 1.79 stars), Positive Care Experience (T1: 27.2%, 4.67), Service Attitude (T3: 17.3%, 1.69), Facility & Environment Quality (T4: 8.1%, 2.73), Registration & Billing Process (T2: 6.9%, 1.83), Surgical & Specialty Care (T5: 5.3%, 4.02), and Inpatient Care Experience (T6: 4.3%, 2.35). In the U.S., the seven dimensions were Overall Positive Care Experience (U2: 26.7%, 4.84), Emergency Care & Wait Time (U1: 23.4%, 2.02), Professional Medical Team (U4: 15.1%, 4.60), Treatment & Pain Management (U5: 12.5%, 2.11), Appointment & Scheduling (U7: 10.4%, 2.00), Clinic Systems & Access (U3: 7.8%, 2.60), and Billing & Insurance Issues (U6: 4.1%, 2.40). Both corpora produced interpretable dimensions spanning clinical care, administrative processes, and interpersonal interactions, though the specific dimensional structures differed by healthcare context (Table 2 and Table 3).

*RQ2: Which dimensions are shared across systems, and which are system-specific?* Cross-lingual semantic mapping of the 14 dimensions identified five universal pairs—four with High similarity (emergency care, positive care experience, professional medical team, and administrative process) and one with Medium similarity (inpatient/treatment care)—along with four system-specific dimensions that appeared in only one country (Table 4, Figure 2). Together, the five universal dimensions accounted for 74.6% of Taiwanese reviews and 88.1% of U.S. reviews, indicating that core hospital quality concerns are broadly shared across the two systems. The four system-specific dimensions were Taiwan’s Service Attitude and Facility & Environment Quality, and the U.S.’s Billing & Insurance Issues and Clinic Systems & Access.

*RQ3: How do institutional and cultural contexts shape these cross-national patterns?* Two sets of evidence address this question. Rating comparisons across the four High-similarity universal dimensions (Table 6) reveal that three pairs differ significantly but with small to medium effects: Professional Medical Team shows the largest gap (*d* = −0.52, favoring U.S. hospitals), while Emergency Care and Positive Care Experience show small effects (*d* = −0.16 and −0.18); Administrative Process shows no significant difference. These patterns suggest that universal dimensions share similar evaluative valence across systems despite institutional differences. Dimension presence/absence patterns offer further insight. Three system-specific dimensions appear to reflect institutional structures: U.S. Billing & Insurance from multi-payer financing, U.S. Clinic Systems from integrated delivery networks, and Taiwan’s Facility & Environment from infrastructure strain under high-utilization single-payer access. The fourth, Taiwan’s Service Attitude, appears more closely linked to cultural factors: consistent with research on collectivist cultural orientation and relational expectations [28,29], Taiwanese patients devote a standalone dimension to interpersonal demeanor, whereas U.S. reviews absorb such concerns into broader experience categories.

### 4.2. Theoretical Implications

First, our findings contribute to the long-standing debate about whether service quality is universal or context dependent. The evidence supports a hybrid interpretation. Patients in both countries consistently highlight core concerns such as emergency responsiveness, clinical competence, and administrative efficiency, demonstrating universal evaluative anchors in hospital care. However, each healthcare system also generates unique dimensional structures shaped by institutional arrangements and cultural values. This pattern challenges the common assumption in frameworks such as SERVQUAL [1] and Dagger et al. [6] that dimensional structures can be applied uniformly across contexts. Instead, the findings suggest that service quality frameworks must differentiate between broad evaluative categories and their local manifestations.

Second, the cross-national comparison sheds light on how institutional and cultural forces differently shape service quality perceptions. Institutional factors appear to play a more prominent role in determining which evaluative domains emerge. Three of the four system-specific dimensions appear to reflect healthcare system structure (U.S. Billing and Insurance Issues from multi-payer financing; U.S. Clinic Systems from fragmented integrated networks; Taiwan Facility and Environment from high-utilization infrastructure strain), while one more clearly reflects cultural norms (Taiwan Service Attitude). The emergence of Service Attitude as a standalone Taiwan dimension—the third-largest dimension and the lowest-rated—is consistent with cross-cultural service research showing that customers from Asian cultural backgrounds place greater weight on personalized service dimensions in service encounters [32]. This aligns with person-centred care research emphasizing respect and relational quality as foundational to patient experience, though existing evidence derives predominantly from Western high-income contexts [33]. This finding underscores that cultural values shape not only the interpretation of shared dimensions but can also generate entirely distinct evaluative domains. However, because institutional and cultural factors were not formally modeled or disentangled in this study, the relative contributions remain suggestive rather than conclusive.

Third, the findings have implications for how patient experience is measured in CQI programs. Structured instruments such as HCAHPS and PREMs benchmark patients against predefined domains, but they are less able to signal new or system-specific concerns—U.S. billing and insurance burden, or Taiwan’s service attitude and facility strain—until those concerns accumulate in administrative data. Inductive analysis of unsolicited patient narratives complements structured measurement by providing a monitoring signal that is sensitive to locally salient quality domains as they evolve. Integrating this inductive channel into CQI systems would allow hospitals and health systems to detect emerging patient-experience problems earlier and to align improvement priorities with the concerns patients spontaneously voice, rather than only those the instrument was designed to capture.

### 4.3. Practical Implications

Our findings identify shared and system-specific CQI priorities for hospital leaders in both countries. Emergency care and administrative efficiency emerge as universally low-rated improvement domains. Although the sources of administrative friction differ—on-site queuing in Taiwan and appointment or scheduling difficulties in the United States—the monitoring signal is consistent across systems and points to a shared CQI target: simplifying and coordinating the patient-facing administrative processes that frame every clinical encounter.

In Taiwan, three actionable domains stand out: emergency responsiveness, service attitude, and facility strain. These call for CQI interventions focused on patient-flow management, frontline staff training and support, and infrastructure investment. In the United States, CQI efforts should prioritize billing and insurance processing and care coordination across multi-site systems; streamlining administrative pathways and improving cost and authorization transparency could substantially reduce the recurring patient frustration captured in our U.S.-specific dimensions.

Cross-national learning offers additional improvement opportunities, consistent with comparative health systems evidence that uses international benchmarking to highlight areas for reform [34]. U.S. hospitals’ consistently higher ratings on specialized surgical and procedural care (Cohen’s *d* = −0.52) suggest that Taiwanese hospitals could benefit from studying U.S. practices in complex procedural pathways—for instance, team-based surgical briefing protocols widely adopted after the WHO Surgical Safety Checklist [35] could inform Taiwanese medical centers seeking to improve procedural coordination ratings. Conversely, U.S. policymakers may draw on Taiwan’s experience to examine how simplified financing structures reduce the billing and insurance burden that currently dominates U.S. patients’ quality evaluations [22]; Taiwan’s single-payer model eliminates the prior-authorization and surprise-billing complaints that constitute an entire U.S.-specific dimension.

### 4.4. Limitations and Future Research

This study has several limitations that provide opportunities for future research.

First, the use of Google Maps reviews introduces multiple sources of bias. Online review platforms attract a self-selected subset of users who tend to report more extreme experiences [36,37]. Elderly patients, lower-income populations, and individuals with limited digital literacy are likely underrepresented, which may skew the dimensional structure toward concerns more salient to younger, digitally active demographics. Additionally, restricting the analysis to Google Maps alone means that other platforms—such as Yelp or Healthgrades in the United States—may capture different patient populations and concerns, and cross-platform replication would strengthen the generalizability of the dimensional structures identified here. Second, LDA topic modeling has inherent methodological limitations. The bag-of-words representation discards word order and syntactic structure, potentially merging semantically distinct phrases. The number of topics K must be specified a priori, and while we evaluated multiple candidates using coherence metrics and qualitative inspection, topic model selection inevitably involves interpretive judgment. LDA also assumes topic independence and cannot capture sentiment polarity within topics—a single topic may contain both positive and negative reviews about the same service domain.

Third, the dataset is cross-sectional, limiting causal inference; cultural versus institutional explanations cannot be fully disentangled without direct measurement of these constructs; and treating each country as homogeneous obscures regional and organizational variation.

Methodologically, applying alternative text-analytic approaches—such as transformer-based topic models (e.g., BERTopic), aspect-based sentiment analysis, or supervised classification—would test whether the dimensional structures identified here remain stable under different analytic assumptions. Future research could also broaden the evidence base by integrating online reviews with survey and interview data, expanding samples to include community hospitals and additional health systems, and employing longitudinal or quasi-experimental designs to examine how service quality dimensions evolve in response to reforms.

## 5. Conclusions

Applying separate language-specific LDA topic models to 5007 Taiwanese and 3240 U.S. Google Maps hospital reviews, we identified seven service quality dimensions in each country. Five were cross-nationally shared, accounting for 74.6% of Taiwanese and 88.1% of U.S. reviews, while four system-specific dimensions reflected distinct institutional and cultural contexts. The pattern of system-specific dimensions suggests that institutional arrangements may play a more prominent role than cultural values in shaping which evaluative domains emerge, though both forces contribute and their relative influence requires further investigation with direct measurement. These institutional and cultural differences create or eliminate entire evaluative domains that predefined patient-experience instruments cannot anticipate, suggesting that inductive analysis of unsolicited patient narratives can serve as a scalable, context-sensitive monitoring signal alongside HCAHPS, PREMs, and similar structured measures. For CQI, the implication is practical: benchmark against universal priorities, but calibrate system-specific interventions to the evaluative domains that each healthcare context actually generates.

## Figures and Tables

**Table 1:** Topic Coherence (c_v_) for Candidate Models (K = 2–10).

K	Taiwan c_v_	U.S. c_v_
2	0.464	0.398
3	0.456	0.437
4	0.423	0.413
5	0.433	0.404
6	0.430	0.409
7	0.418	0.382
8	0.394	0.395
9	0.363	0.383
10	0.395	0.362

Note: c_v_ = coherence score, with higher values indicating greater semantic coherence.

**Table 2:** Taiwan Hospital Service Quality Dimensions (N = 5007).

Topic	Dimension Name	n	Proportion	Avg Rating	Top-10 Keywords (English Translation)
T1	Positive Care Experience	1360	27.2%	4.67	physician, nurse, professional, thank you, gratitude, doctor, kind, medical, patient, staff
T2	Registration & Billing Process	343	6.9%	1.83	time, registration, consultation, appointment, payment, medication pickup, afternoon, clinic
T3	Service Attitude	866	17.3%	1.69	attitude, patient, nurse, staff, not, blood draw, examination, registration, impatient
T4	Facility & Environment Quality	407	8.1%	2.73	time, parking lot, convenient, consultation, waiting, parking, elevator, center, flow
T5	Surgical & Specialty Care	266	5.3%	4.02	physician, surgery, thank you, operation, surgical dept, nurse, orthopedics, treatment
T6	Inpatient Care Experience	218	4.3%	2.35	ward, hospitalization, family, patient, nurse, discharge, doctor, relatives, surgery
T7	Emergency Medical Services	1547	30.9%	1.79	doctor, we, emergency, patient, know, not, issue, nurse, examination, one

**Table 3:** U.S. Hospital Service Quality Dimensions (N = 3240).

Topic	Dimension Name	n	Proportion	Avg Rating	Top-10 Keywords
U1	Emergency Care & Wait Time	759	23.4%	2.02	room, nurse, hour, patient, time, waiting, emergency, care, came, never
U2	Overall Positive Care Experience	865	26.7%	4.84	nurse, staff, care, great, thank, best, doctor, amazing, everyone, professional
U3	Clinic Systems & Access	253	7.8%	2.60	clinic, care, patient, cleveland, service, mayo, family, staff, father, hospital
U4	Professional Medical Team	489	15.1%	4.60	care, staff, surgery, experience, medical, nurse, patient, procedure, well, professional
U5	Treatment & Pain Management	404	12.5%	2.11	pain, day, could, help, even, time, never, back, nurse, life
U6	Billing & Insurance Issues	134	4.1%	2.40	bill, year, billing, surgery, clinic, phone, pay, insurance, system, patient
U7	Appointment & Scheduling	336	10.4%	2.00	appointment, care, time, test, month, patient, call, even, back, people

**Table 4:** Cross-Lingual Semantic Mapping of Universal Dimensions.

Universal Dimension	Taiwan Topic	U.S. Topic	Similarity
Emergency Care	T7 (30.9%, 1.79)	U1 (23.4%, 2.02)	High
Positive Care Experience	T1 (27.2%, 4.67)	U2 (26.7%, 4.84)	High
Professional Medical Team	T5 (5.3%, 4.02)	U4 (15.1%, 4.60)	High
Administrative Process	T2 (6.9%, 1.83)	U7 (10.4%, 2.00)	High
Inpatient/Treatment Care	T6 (4.3%, 2.35)	U5 (12.5%, 2.11)	Medium

Note: Format shows topic ID (proportion, average rating). See Table 2 and Table 3 for complete dimension descriptions.

**Table 5:** System-Specific Service Quality Dimensions.

Country	Dimension	n	Proportion	Rating	Institutional/Cultural Factor
Taiwan Only	Service Attitude (T3)	866	17.3%	1.69	Cultural emphasis on interpersonal respect
Taiwan Only	Facility & Environment (T4)	407	8.1%	2.73	Infrastructure strain from high utilization
U.S. Only	Clinic Systems & Access (U3)	253	7.8%	2.60	Large multi-site integrated systems
U.S. Only	Billing & Insurance (U6)	134	4.1%	2.40	Multi-payer financial complexity

**Table 6:** Cross-National Rating Comparisons.

Universal Dimension	Taiwan (%, Rating)	U.S. (%, Rating)	*t*	*p*	Cohen’s *d*
Emergency Care	30.9%, 1.79	23.4%, 2.02	−3.68	<0.001	−0.16
Positive Care Experience	27.2%, 4.67	26.7%, 4.84	−4.04	<0.001	−0.18
Professional Medical Team	5.3%, 4.02	15.1%, 4.60	−6.69	<0.001	−0.52
Administrative Process	6.9%, 1.83	10.4%, 2.00	−1.57	0.117	−0.12

Note: All *t*-tests employed Welch’s correction for unequal variances. Cohen’s *d* interpretation: 0.2 (small), 0.5 (medium), 0.8 (large).

## Data Availability

The raw review data are not publicly available due to Google Maps platform terms of service. Derived aggregate data supporting the findings of this study are available from the corresponding author upon reasonable request.
